# Bibliography

**Published:** 1885-12

**Authors:** 


### BIBLIOGRAPHY.

### BOOKS RECEIVED.

*A Practical Treatise on Diseases of Children*, by Alfred Vogel, M. D., Professor of Clinical Medicine in the University of Dorpat, Russia. Translated and edited by H. Raphael, M. D., formerly House Surgeon to Bellevue Hospital, Physician to the Eastern Dispensary for Diseases of Children, etc., etc. Third American from the Eighth German Edition. Revised and enlarged; illustrated by six lithographic plates. New York, D. Appleton & Co., 1, 3 and 5 Bond street, 1885; pages 604, cloth.

This is indeed a valuable addition to the literature of Pediatrics. The Germans, excelling in their attention to minutia in most else, are recognized as authority on all that pertains to diseases of children and therapeutics. Dr. Vogel's Treatise on Diseases of Childen rapidly ran through eight editions in Europe, and was translated into every living language on the globe. In this the latest edition (3rd American) much has been added to the chapters on *Artificial Nutrition*, a subject of deep interest to the practitioner; on Difficulties of Dentition, and on Nervous Diseases of Children. The very important question in Pediatrics, concerning the presumed danger of transmission of tuberculosis directly from the cow to the child by means of the milk, has been fairly discussed. Many of the newer remedies, such as apomorphia, salicylic acid, encalyptus, have been mentioned, and their uses hypodermically have been discussed, and their advantages and disadvantages have been clearly pointed out. This alone should be worth the price of the book, as the treatment of diseases of children is too much after the stereotyped fashioned of the last century.

This work, Lusk's Midwifery and Weber's Nervous Diseases —all from the great publishing house of APPLETON—are uniform in a handsome brown muslin binding.

*Hand Book of Therapeutics*, H. VonZiemssen's. We have received Vol. III of this valuable series from the publishers Messrs. Wm. Wood & Co., N. Y.

This volume treats of "The Therapeutics of the Repiratory Organs," and is of course uniform with the set—to be completed in seven volumes. Extended notices have been given of the first two volumes. The third is in keeping with the general plan of the work, and is characterized by the same pains-taking, and thorough discussion of every subject handled in connection with Diseases of the Organs of which it treats. When completed, this work alone will constitute a small library of information on every branch of general practice; equal to a Treatise on Practice of Medicine, Therapeutics, Hygiene and Dietetics combined, with Physiology thrown in.

*Hand Book of Diseases of the Skin.*—Edited by H. v. Zeimssen, M. D., Prof. Clinical Medicine in Munich, editor of *Zeimssen's Cyclopædia of the Practice of Medicine.* Illustrated with 80 wood engravings and color plates; p. 654. New York: Wm. Wood & Co. 1885.

This volume should have been vol. 9 of the Great Cyclopædia of Practice, but as it was not published in Germany until long after all the rest of the series were out, and the Cyclopædia had been so long in going through the press that the publishers, being pressed by subscribers, perhaps, determined to close it up without the volume on skin diseases. Some subscribers, however, complained, and Wood & Co. can't stand that, you know, although it was no fault of theirs; so just as soon as it appeared in Germany, Wood & Co. caused its translation, and got it out in a style different from that of the other volumes—in very handsome embossed cloth—and *made subscribers a present of it!* Each volume has inscribed on the back "PRESENTED WITH THE COMPLIMENTS OF THE PUBLISHERS."

The idea of a publishing house getting out a work of this kind, in elaborate and very expensive style, to GIVE AWAY by the thousands, is certainly without precedent in the book-printing busiuess; and no house but this, whose head entertained at his own expense, in princely style, the entire representation at the annual convention of the National Medical Association, in New York, a few years ago, would ever have thought of doing so. The medical profession certainly have cause to remember, most gratefully, the house of Wm. Wood & Co., of whose princely generosity they have more than once been the recipients.

To speak of the book itself would be but a repetion of what has been said of the antecedent volumes. It is written with that same careful, almost painful attention to the minutest details; is elaborate and thorough in every department, and brings the reader up abreast with the most advanced views as to etiology, pathology and therapeutics of that troublesome class of diseases—diseases of the skin and hair.

It is the handsomest book in our library, and, outside of its intrinsic value, has a value that only attaches to a *souvenir* from a friend.

*Index Catalogue of the Library of the Surgeon General's Office, U. S. Army*:—Authors and Subjects; Vol. I. to VI;—Heastie—Insfelt; Washington; Government Printing Office, 1884.

These volumes are part—the completed part of the Herculean labor undertaken by Dr. J. S. Billings, Surgeon U. S. A., in charge of the Library of the Surgeon General's Office. When it is completed it will constitute a monument to his ability and painstaking patient labor—*con amore*. It is a work necessary to be done; otherwise the mountain of literature—sands on the shores of the medical world, would be a useless, meaningless mass. It is the *key* by which the student and bibliographer can unlock those treasures, and turn them to use. In this library are preserved copies of every book, pamphlet, and reprint on every subject connected with the science and art of medicine and surgery in all their branches—*buried* as it were; and without this key, which Dr. Billings is now preparing, they would be, as said, as meaningless and as useless as are the *strata* which mark the history of the earth's formation, to the savage. The medical profession then of the present day not alone will be indebted to Dr. Billings; he is now doing a benefaction which will be felt and appreciated many generations hence, unless, like the great Alexandrian library, it should be swept from existence.

We take it as quite a compliment to receive those valuable volumes, and take this occasion to express our acknowledgement of the courtesy.

*The Science and Art of Midwifery*, by Wm. Thompson Lusk, A. M., M. D., etc. New edition, revised and enlarged, with numerous illustrations. Pages, 720. New York: D. Appleton & Co. 1885.

Within a short period this popular work has run through several editions. The present edition has engrafted into it much that has been learned since the appearance of the first—for in no department of medical study has more progress been made than in obstetrics. Especially the learned author draws attention to the triumph of modern doctrines concerning the nature of puerperal fever—that horror of the lying-in-room, owing to the application of Listers' heaven-inspired teachings, to the hygiene of the parturient state—as they have been applied in every other part of the domain of medicine and surgery. The merest tyro can appreciate what antisepsis has done already for the lying-in-room, as well as for surgery. To such an extent is antisepsis carried in hospitals that the author says "it is now a question whether forlorn women, confined in properly conducted" maternities "are not actually safer in child-bed than the fortunate class in their own homes." Fortunately—thanks to these very teachings—the better class enjoy the blessings of antisepsis as well as hospital patients.

The volume, as perfected, and brought up to the latest teachings, is what the author says he aimed to make it, "A safe guide to the practice of midwifery, and a useful book of reference for those who wish to investigate modern teachings at their source." This edition bears date May 23, 1885 It will constitute both a valuable and ornamental addition to any physician's library.

